# Deep Learning Analysis of Localized Interlayer Stacking Displacement and Dynamics in Bilayer Phosphorene

**DOI:** 10.1002/adma.202416480

**Published:** 2025-03-03

**Authors:** Kihyun Lee, Sol Lee, Yangjin Lee, Kwanpyo Kim

**Affiliations:** ^1^ Department of Physics Yonsei University Seoul 03722 South Korea; ^2^ Center for Nanomedicine Institute for Basic Science Seoul 03722 South Korea; ^3^ Department of Energy Science Sungkyunkwan University Suwon 16419 South Korea

**Keywords:** deep learning analysis, edge reconstruction, in situ TEM analysis, phosphorene, stacking order

## Abstract

The interlayer displacement has recently emerged as a crucial tuning parameter to control diverse physical properties in layered crystals. Transmission electron microscopy (TEM), an exceptionally powerful tool for structural analysis, directly observes the interlayer stacking and strain fields in various crystals. However, conventional analysis methods based on high‐resolution phase‐contrast TEM images are inadequate for recognizing spatially varying unit‐cell patterns and their associated structure factors, hindering precise determination of interlayer displacements. Here, a deep learning‐based analysis is introduced for atomic resolution TEM images, enabling unit‐cell pattern recognition and precise identification of interlayer stacking displacement in bilayer phosphorene. The deep learning model applied to bilayer phosphorene accurately determines stacking displacement, with an error level of 3.3% displacement within the unit cell and a spatial resolution approaching the individual unit‐cell level. Additionally, the model successfully processes a large set of in situ TEM data, capturing spatially varying, time‐dependent interlayer displacement dynamics associated with edge reconstruction, demonstrating its potential for processing large‐scale microscopy datasets.

## Graphical abstract

Interlayer displacement plays a crucial role in tuning properties of layered crystals, yet its precise and efficient determination remains challenging. A deep learning‐based analysis is presented for atomic‐resolution phase‐contrast transmission electron microscopy (TEM) images, accurately identifying stacking displacement in bilayer phosphorene with near unit‐cell resolution. The model processes large‐scale in situ TEM data, capturing time‐dependent displacement dynamics linked to edge reconstruction.

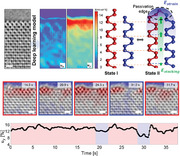

## 1 Introduction

In van der Waals (vdW) layered materials, interlayer displacements caused by sliding have become a critical mechanism for modulating electrical properties, magnetism, ferroelectric characteristics, and phase transitions within these systems.^[^ ^1^, ^2^, ^3^, ^4^, ^5^, ^6^, ^7^, ^8^, ^9^ ^]^ For example, crystals like MoS_2_, WS_2_, and In_2_Se_3_ demonstrate electric polarization that depends on stacking, with active switching capabilities.^[^ ^7^, ^10^, ^11^, ^12^, ^13^, ^14^, ^15^ ^]^ Similarly, metal trihalides such as CrI_3_ display switching between ferromagnetic and antiferromagnetic properties that also depend on how they are stacked.^[^ ^16^, ^17^, ^18^, ^19^, ^20^, ^21^, ^22^ ^]^ In twisted vdW crystals, the moiré pattern creates a superstructure featuring diverse local stacking arrangements, which results in strongly correlated phenomena, such as superconductivity,^[^ ^1^, ^2^, ^23^, ^24^ ^]^ particularly pronounced at low twist angles. In particular, in‐plane atomic reconstruction results in complex stacking configurations for low twist angles.^[^ ^25^, ^26^, ^27^, ^28^, ^29^ ^]^ Therefore, the identification and characterization of stacking configurations are essential for elucidating the relationship between structural arrangements and their corresponding physical properties.

Techniques for identifying local stacking configurations in vdW crystals encompass various microscopic methods, including atomic force microscopy,^[^ ^28^, ^30^ ^]^ piezo force microscopy,^[^ ^31^, ^32^ ^]^ Raman spectroscopy,^[^ ^33^, ^34^, ^35^ ^]^ infrared spectroscopy,^[^ ^36^, ^37^, ^38^ ^]^ and dark field (DF) imaging methods in transmission electron microscopy (TEM).^[^ ^27^, ^29^, ^39^ ^]^ These methods generally suffer from limited spatial resolution, which makes analyzing stacking configurations at the nanometer scale challenging. To investigate the stacking arrangement with nanometer or atomic resolution, techniques such as atomic‐resolution scanning TEM (STEM),^[^ ^40^ ^]^ four dimensional STEM (4D‐STEM),^[^ ^41^, ^42^, ^43^ ^]^ and scanning tunneling microscopy (STM)^[^ ^44^, ^45^ ^]^ are used. Although these techniques are well‐suited for studying static systems, their limited temporal resolution renders them less effective for investigating dynamic behaviors in stacking configurations, such as those driven by the movement of interlayer‐displacement solitons.^[^ ^25^, ^29^, ^46^, ^47^, ^48^ ^]^ In contrast, phase‐contrast TEM imaging provides higher temporal resolution with comparable spatial resolution. However, traditional methods for analyzing phase‐contrast TEM images, such as geometric phase analysis (GPA)^[^ ^49^, ^50^ ^]^ and peak pair analysis (PPA),^[^ ^51^ ^]^ often fail to accurately identify stacking configurations (Note S1 and Figure S1, Supporting Information). This highlights the necessity for developing a new, facile data processing method for phase‐contrast TEM images.

Recent innovations, particularly in the field of artificial intelligence, have made feature recognition in TEM data substantially more effective. The use of convolutional neural networks (CNN) has greatly facilitated diverse structural analyses in TEM and STEM images,^[^ ^52^, ^53^, ^54^, ^55^, ^56^, ^57^, ^58^, ^59^, ^60^, ^61^, ^62^ ^]^ which include evaluating point defects, phase structure transitions, and data denoising. Here, we develop a deep learning‐based approach to analyze atomic‐resolution TEM images, which is capable of recognizing unit‐cell patterns and accurately identifying interlayer stacking displacement in bilayer phosphorene. This method overcomes the limitations of traditional techniques such as GPA or PPA in identifying interlayer stacking displacements from phase‐contrast TEM images. The deep learning model delivers highly precise detections of stacking displacements, with an error level of only 3.3% within the unit cell, and achieves spatial resolution at the scale of individual unit cells. Additionally, this model has been successfully implemented on a vast collection of in situ TEM data of bilayer phosphorene, capturing the spatially varying and time‐dependent dynamics of interlayer displacement associated with edge reconstruction, thus demonstrating its potential for processing large‐scale microscopy datasets.

## 2 Results and Discussions

We selected phosphorene,^[^ ^63^, ^64^, ^65^, ^66^ ^]^ a 2D vdW material known for its excellent electrical and optoelectrical characteristics,^[^ ^67^, ^68^, ^69^, ^70^, ^71^, ^72^ ^]^ as a model system to investigate stacking displacements and their identification. Phosphorene's puckered, highly anisotropic in‐plane structure differs significantly from conventional hexagonal 2D crystals,^[^ ^68^, ^73^, ^74^, ^75^, ^76^, ^77^, ^78^, ^79^, ^80^ ^]^ and the preferred interlayer displacement directions and patterns have not been extensively studied. Owing to its unique structural features and strong interlayer coupling,^[^ ^81^ ^]^ phosphorene's electrical bandgap varies significantly with the number of layers, from 0.3 eV in bulk to 1.8 eV in a monolayer.^[^ ^73^, ^76^ ^]^ Previous studies have confirmed that the AB stacking, with its alternating stacking configuration, is the energetically preferred configuration.^[^ ^82^, ^83^, ^84^ ^]^

**Figure 1:**
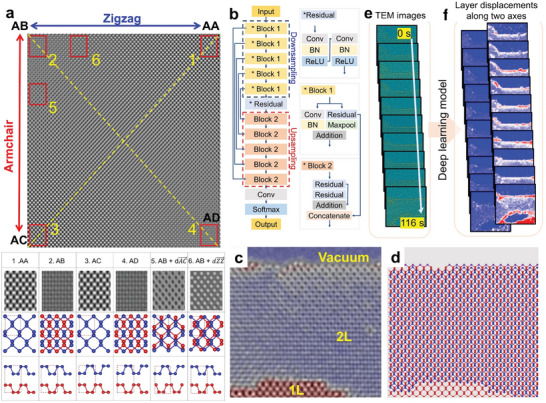
Fully convolutional network (FCN) for unit‐cell pattern and interlayer displacement recognition in bilayer phosphorene. a) Phase‐contrast TEM simulation image of bilayer phosphorene, displaying various stacking configurations under a defocus value of 14 nm. The stable AB stacking configuration is depicted in the top‐left corner. Various stacking configurations (AA, AC, AD, and other intermediate configurations) can result from relative in‐plane displacements along the armchair and/or zigzag lattice directions. The table at the bottom showcases zoomed‐in TEM simulation images from different areas of the structure, marked by red rectangular boxes. b) Structure of the FCN model. c) An exemplary experimental TEM image of bilayer phosphorene. d) Atomic model derived from panel c. e) In situ time‐series TEM images provided as input for the model. f) Output results displaying the identification of interlayer displacements.

The interlayer displacement in bilayer phosphorene can be discerned through TEM or STEM imaging. **Figure** **1**a shows simulated TEM images for various interlayer stacking displacements. Starting with the preferred AB stacking (top‐left corner), different stacking configurations were generated by relative interlayer displacements along the armchair (*u*_armchair_) or zigzag (*u*_zigzag_) lattice directions. A displacement by half the unit‐cell along the armchair (*u*_armchair_ =  0.5) or zigzag direction (*u*_zigzag_ = 0.5) changes the stacking from AB to AC or AA, respectively.^[^ ^66^ ^]^ Each stacking configuration displays a distinct phase‐contrast TEM image pattern. For example, AB stacking yields a rectangular pattern, whereas the AA stacking configuration, where the top and bottom layers are aligned, shows a diamond pattern (Figure 1a). Other intermediate stacking configurations also produce unique TEM image patterns. These stackings have diverse physical properties, such as interlayer distance, electrical bandgap, and phonon modes, as summarized in Table S1 (Supporting Information).^[^ ^85^, ^86^ ^]^ It is crucial to note that top‐view TEM images are not sensitive to out‐of‐plane atomic positions, meaning different stacking structures (e.g., AB and AD configurations) appear identical in these images. Consequently, the simulated images in Figure 1a are doubly degenerate, and the structures can only be differentiated in half of the regions.

We developed a deep learning model capable of quantitatively analyzing stacking displacements in bilayer phosphorene. Figure 1b displays the architecture of our model, a residual convolutional neural network (ResUNet), designed for image segmentation.^[^ ^53^, ^60^ ^]^ The encoding/decoding process occurs as each block propagates to the next kernel, with data from the encoding phase being efficiently extracted by the decoding kernel.^[^ ^87^, ^88^ ^]^ Figure 1c,d shows input examples of bilayer phosphorene TEM image and corresponding atomic structure model, respectively. To fully harness the deep learning model's power for processing large datasets, in situ TEM time series data were analyzed using the model. Figure 1e,f illustrate the input of experimental in situ TEM time‐series into the model, with the resulting video revealing the interlayer displacement along major/minor axes in phosphorene. We will explain later that the major axis corresponds to the armchair direction, and the minor axis to the zigzag direction in our study.

**Figure 2:**
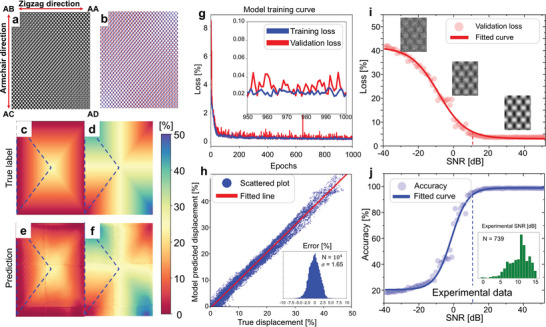
Model training and performance validation using simulated phase‐contrast TEM images at various noise levels. a) TEM simulation image of bilayer phosphorene, showcasing different stacking configurations under a defocus value of 14 nm and b) the corresponding atomic model structure. c,d) True labels indicating interlayer displacements along major and minor axes in the atomic model. e,f) Model predictions for interlayer displacements along major and minor axes. g) Training and validation loss curves plotted against the number of epochs. The inset shows the loss curves around 1000 epochs. h) Scatter plot comparing model predictions with true labels. The inset displays an error histogram between the true and predicted displacement values. i,j) Validation loss and accuracy curves plotted against the signal‐to‐noise ratio in simulated images. The inset in panel j illustrates an SNR histogram of analyzed experimental images.

We generated a total of 40960 simulated TEM images and trained the deep learning model with these images. Detailed methods of simulation and training are provided in the Experimental Section, Note S2, and Figures S2–S5 (Supporting Information). Notably, intensity patterns in phase‐contrast TEM imaging are sensitive to defocus values, which can even invert the positions of bright and dark intensities (Figure S4, Supporting Information). The nature of phase contrast imaging presents a challenge in model training when using actual displacements along the zigzag and armchair directions as labels. To overcome this, we labeled the images based on major and minor displacement axes, which allowed us to determine the magnitudes of the two displacement components without specifying whether they corresponded to armchair or zigzag directions (Figure S2, Supporting Information). **Figure** **2**a depicts an exemplary input TEM image, while Figure 2b displays the corresponding atomic model, highlighting various local stacking configurations. Due to gradual changes in interlayer displacements, different locations exhibit varying displacement values along two axes, as depicted in Figure 2c,d. The area inside the dashed blue triangle in Figure 2c–f represents the configuration space where our identification remains distinct, as explained in Supporting Information. Figure 2e,f illustrates the model's predictions, confirming that the model accurately captures the systematic displacement behaviors within the structure.

Figure 2g displays the training and validation loss curves from the model training, showing rapid learning during the initial 50 epochs. After approximately 100 epochs, both the training and validation data exhibit minimal errors. Due to the relatively large momentum of the learning rate, some slight noise appears in the validation loss. Thus, we selected the model's parameters from the epoch with the lowest validation loss as the final model. Figure 2h presents a scatter plot comparing the interlayer stacking displacements identified by the trained model with the true displacements. The slope determined through linear regression is 1.02. The inset in Figure 2h shows a histogram of the errors between true labels and predictions. The standard deviation is 1.65%, implying a 95% confidence interval for an error of 3.3% or less.

We evaluated the model's prediction accuracy under various noise levels in TEM images. Figure 2i illustrates the validation loss curve as a function of the signal‐to‐noise ratio (SNR), defined as the ratio of signal variance to noise variance: SNR = $10\log_{10}\left(\frac{\mathrm{variance}\;\mathrm{of}\;\mathrm{signal}}{\mathrm{variance}\;\mathrm{of}\;\mathrm{noise}}\right)\left[\mathrm{dB}\right]$.^[^ ^89^ ^]^ Figure 2j depicts the accuracy curve, considering a displacement magnitude error less than 5% loss as a correct prediction. Fitting this curve to the Boltzmann distribution provides a threshold center of ‐1.38. Our analysis indicates that the average SNR for experimental TEM data is approximately 10.3, which suggests a predicted accuracy of over 90%. To further validate the model's performance, we compared its outcomes with those derived from fast Fourier transform (FFT) analysis of TEM images and calculations of structure factors (Figure S6, Supporting Information).

We note that conventional methods encounter significant challenges in extracting stacking configurations from phase‐contrast TEM images. Common techniques for analyzing strain and dislocations in atomic‐scale images, such as PPA and GPA, are not well‐suited for assessing interlayer displacements. PPA,^[^ ^51^ ^]^ which necessitates precise identification of atomic positions, is generally unsuitable for phase‐contrast TEM images and often struggles to handle small displacement data. GPA,^[^ ^49^, ^50^ ^]^ depending on Fourier transform to extract specific Bragg peaks and dislocations using phase information, proves ineffective in this scenario, as the dominant effect stems from structural interference rather than layer displacements (Note S1 and Figure S1, Supporting Information).

To fully utilize our developed model, we applied the methodology to in situ TEM data of bilayer phosphorene. The observed sample was graphene‐supported bilayer phosphorene, and the graphene signal was selectively removed through masking during FFT analysis of the TEM images. Detailed information on sample preparation and data acquisition is available in the Experimental section. In the in situ TEM data, the bilayer phosphorene undergoes various time‐dependent changes, including edge‐mediated etching, localized stacking displacements due to edge reconstruction, and occasional defect formation in basal planes.^[^ ^83^, ^90^ ^]^ Previous studies have demonstrated that the zigzag‐terminated edge of bilayer phosphorene stabilizes in a self‐passivated configuration with interlayer bonding. This edge reconstruction substantially alters its properties and plays a vital role in the synthesis of phosphorene.^[^ ^90^, ^91^, ^92^, ^93^, ^94^, ^95^, ^96^ ^]^ Additionally, edge reconstruction is expected to cause layer sliding along the armchair lattice direction perpendicular to the edge termination, resulting in localized strain near the edge. By analyzing in situ TEM data, we aim to gain deeper insights into the formation and destabilization of edge states, and the behavior of localized displacements.

**Figure 3:**
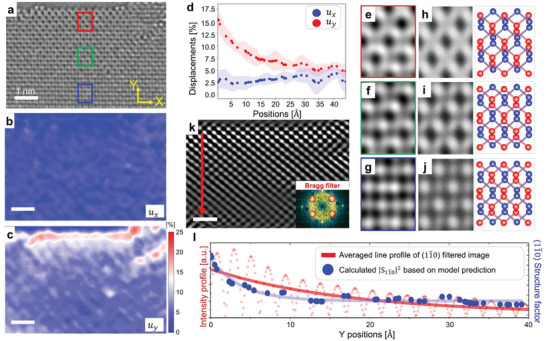
Analysis of stacking displacement localized near the edge of bilayer phosphorene. a) TEM image of the bilayer phosphorene edge. b,c) Color‐coded maps display interlayer displacements along the zigzag (x‐axis) and armchair (y‐axis) lattice directions, respectively. The magnitude of layer displacement is represented by a color gradient from red to blue (scale bar: 1 nm). d) Model‐predicted average interlayer displacements along the zigzag and armchair directions are shown. The x‐axis in the panel indicates the position relative to the edge. e–g) Zoomed‐in experimental TEM images from different sections in panel a. Each image corresponds to rectangular areas with various colors. h–j) Simulated TEM images (defocus: 14 nm) and atomic models based on the predicted interlayer displacements for respective regions. k) Bragg‐filtered TEM image utilizing the ($1\bar{1}0$) peak. The inset illustrates the Fourier transform of the original image with the $(1\bar{1}0$) peak selected (scale bar: 1 nm). l) A comparison of stacking displacement results derived from deep learning and Bragg‐filtering‐based analyses is presented. The average intensity profile (in red) from the Bragg‐filtered images and the calculated $(1\bar{1}0$) structure factor (in blue), obtained from model‐predicted interlayer displacements, are showcased.

**Figure** **3**a presents a snapshot from a TEM image extracted from a Movie S1 (Supporting Information), where the sample's top is terminated with a zigzag lattice direction. By utilizing deep learning processing, we produced a map of the interlayer displacements, as depicted in Figure 3b,c. In this analysis, we designated the major (minor) displacement as the y‐direction (x‐direction), stemming from our understanding that the major displacement occurs perpendicular to the edge termination. The localized displacement near the edge is pronounced in the y‐direction displacement, as illustrated in Figure 3c. Figure 3d displays the average line profile for both x‐ and y‐displacements as a function of distance from the edge to the lower regions of the sample. The interlayer displacement in the x‐direction is minimal, less than 5% of the unit cell. In contrast, the y‐direction shows a substantial localized stacking displacement with a 15% magnitude that rapidly decays away from the edge. Phosphorus atoms have five outer shell electrons and form three interatomic bonds in energetically favorable structures. The maximum local displacement at the reconstructed edge is constrained by edge bonding configurations, and the observed maximum value of approximately 20% is consisted with the previous structural model.^[^ ^90^ ^]^ By fitting this decay to $D\left(y\right)=Ae^{-y/\tau_{s}}$, we extracted the decay parameter, $\tau_{s}=19.2\pm 3.8$ Å. We further validated the model's predicted displacement values by comparing experimental TEM images with simulation images based on the predicted displacements. Figure 3e–g are experimental TEM images magnified from the marked rectangular boxes in Figure 3a. Figure 3h–j present simulated TEM images based on the layer displacements identified by the model at corresponding positions, demonstrating good agreement with the experimental data.

Bragg filtering can also be employed to distinguish different stacking configurations. The ($1\bar{1}0$) peak from the FFT in bilayer phosphorene is sensitive to the stacking relation, as demonstrated in Figure S6 (Supporting Information). Figure 3k displays the ($1\bar{1}0$) peak‐Bragg filtered image of Figure 3a, which allows for a rough estimation of layer displacement based on local intensities. The localized stacking displacement near the edge is also discerned through the Bragg filtering. The red scattered plot in Figure 3l represents the line profile of the Figure 3k image, whereas the red line indicates the intensity value averged over one unitcell length. For comparison, we calculated the local $\left(1\bar{1}0\right)$ structural factors $\sum_{j=1}^{N}f_{j}\left(|\vec{G}|\right)e^{\left[-2\pi i(hx_{j}+ky_{j}+lz_{j})\right]}$ from the model‐predicted layer displacement values, shown as blue data in Figure 3l. The slight discrepancy observed near the edge stems from the different spatial resolutions of the two methods. The analysis of a Bragg peak signal inherently assumes a periodic structure larger than a single unit cell, and the Bragg filtering process inherently suffers from limited spatial resolution. We confirm that the model‐based analysis provides significantly enhanced spatial resolution with a FWHM of 3.7 Å, approximately the size of a unit‐cell (see Note S4 and Figure S7, Supporting Information).

**Figure 4:**
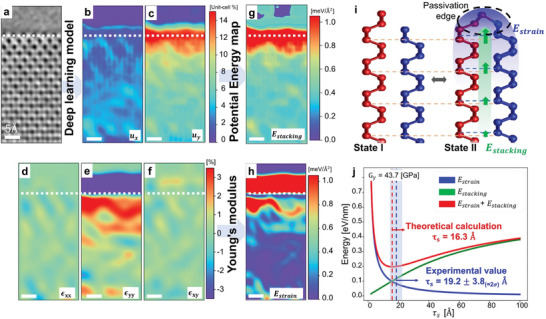
Energy competition between interlayer stacking and in‐plane strain energies near the reconstructed edge. a) TEM image of a zigzag‐terminated reconstructed bilayer phosphorene edge. b,c) Deep learning‐derived interlayer displacement fields from panel a, showing the sub‐unit cell displacement along the x and y axes, respectively. d–f) Calculated strain maps based on the displacement fields. g) Calculated interlayer stacking energy map using model‐predicted displacement fields. h) Calculated in‐plane strain energy map is depicted. All scale bars are 5 Å. i) A schematic illustrating the competition between interlayer stacking energy and strain energies near edge reconstruction. The pristine, unreconstructed edge is labeled as State I, while the reconstructed, self‐passivated edge is referred to as State II. j) Strain, stacking, and total energy variations are shown as a function of the decay parameter (τ_s_) in a simple decaying model.

The localized edge stacking displacements and their decay behavior can be rationalized from an energetics perspective, where the main contributing energy terms are the interlayer stacking energy (*E*_stacking_) and the in‐plane strain energy (*E*_strain_). *E*_stacking_ represents the energy associated with interlayer stacking modification relative to the AB stacking configuration, while *E*_strain_ denotes the energy penalty arising from structural deformation induced by in‐plane strain near reconstructed region. **Figure** **4**a–c showcase a cropped TEM image and model‐predicted layer displacements *u_x_* and *u_y_* in the corresponding region. The in‐plane strains are derived by differentiating these displacements $\left[\in_{xx}=\frac{du_{x}}{dx},\;\in_{yy}=\frac{du_{y}}{dy},\;\mathrm{and}\;\in_{xy}=\frac{1}{2}\left(\frac{du_{x}}{dy}+\frac{du_{y}}{dx}\right)\right]$, as illustrated in Figure 4d–f. Utilizing the potential energy surface (PES) as a function of displacements^[^ ^82^ ^]^ and the Young's modulus of phosphorene,^[^ ^97^ ^]^ we calculated the local *E*_stacking_ and *E*_strain_, presented in Figure 4g,h, respectively. Assuming a simple decay model expressed as $D\left(y\right)=Ae^{-y/\tau_{s}}$, the stacking energy and strain energy are proportional and inversely proportional to the spatial decaying parameter τ_s_, respectively:

(1) $$\Delta E_{\mathrm{total}}=E_{\mathrm{stacking}}+E_{\mathrm{strain}}\;\approx \frac{C_{1}A^{2}}{2}\tau_{s}+\frac{E_{y}A^{2}}{4\tau_{s}}$$

where *C*_1_ and *E_y_* represent the amplitude of the PES and the Young's modulus in the armchair direction, respectively. A detailed description of this analysis can be found in Note S5 and Figure S8 (Supporting Information). Figure 4j shows that the decay parameter is determined by the competition between stacking and strain energy. When τ_s_ is large, an energy penalty arises from the stacking energy component, while a small τ_s_ results in instability from strain energy. The energetically preferred τ_s_ was calculated to be 16.3 Å, which corresponds closely with the experimentally observed value of $\tau_{s}=19.2\pm 3.8$ Å, as depicted in Figure 4j.

We now address the time‐dependent behaviors of phosphorene edges under e‐beam exposure. In the in situ TEM data, bilayer phosphorene exhibits various changes, including edge‐mediated etching, localized stacking displacements due to edge reconstruction, and occasional defect formation in basal planes. In situ time‐series TEM images were analyzed using a deep learning model, revealing time‐dependent interlayer displacements with a temporal resolution of 6 frames per second. Particularly, the deep learning analysis successfully identified slight stacking displacements associated with edge reconstruction, enabling differentiation between State I (pristine bilayer phosphorene edge without reconstruction) and State II (reconstructed, self‐passivated edge state). The e‐beam acts as an energy source that induces the transition between these states,^[^ ^98^ ^]^ through formation and dissociation of interlayer bonding at the edge, as illustrated in Figure 4i.

**Figure 5:**
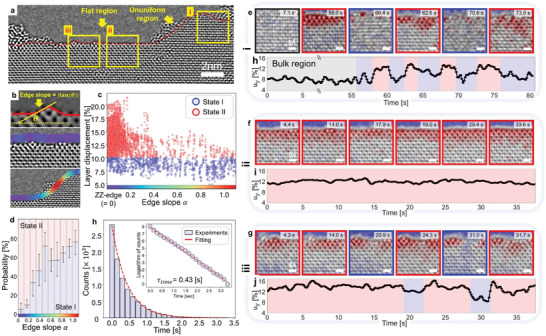
Position‐dependent, dynamical behavior of localized interlayer displacement near the edge. a) TEM image of bilayer phosphorene from a time‐series set, displaying various edge structures. The edge location is marked with dashed red line. The yellow squares highlight the regions analyzed in panels (e–g). b) Zoomed‐in TEM image near the edge with the edge slope overlaid. The local edge slope (color‐coded) is calculated as tan θ. c) Identified interlayer displacements near the edge, as a function of the local edge slope, derived from the time‐series TEM data. An interlayer displacement value exceeding 10% is classified as the reconstructed edge, State II (in red). d) Occupancy of states I and II, correlated with the local edge slope. e–g) TEM time series for different regions of the sample indicated in panel (a). The time series in panel e) illustrates the etching and initial formation of edge reconstruction. Panel f) depicts the behavior of an atomically straight, perfectly reconstructed edge. Panel g) reveals the dynamic behavior of a reconstructed edge with a local defect. Blue and red colors indicate states I and II, respectively. h) Lifetime histogram of State I in region (g). The lifetime is calculated based on the maximum number of frames during which State I persists before transitioning to State II.

The stability and reconstruction behaviors of edges are highly influenced by their terminations. We outline the edge termination with a red line and identified its local slope α  = tan θ, as depicted in **Figure** **5**a,b. Here, α = 0 denotes a zigzag edge termination, with α indicating the deviation from this termination. Figure 5c illustrates the identified local displacement *u_y_* near the edge termination as a function of the corresponding slope α from our data. Assuming the edge with *u_y_* ≥ 10% represents State II, the probability of each state, based on the edge slope, was calculated, as shown in Figure 5d. This behavior, dependent on edge termination, conforms to our understanding that State II only occurs with zigzag termination. Conversely, edges with irregular shapes or non‐zero α cannot facilitate edge reconstruction, resulting in a State I edge.

An extensive analysis of different segments of edge termination reveals insights into the initial formation, stability, and destabilization of State II edges under e‐beam irradiation. Figure 5e–g display time‐series TEM images with the identified displacement *u_y_* overlaid on various regions of the sample (yellow squares in Figure 5a). The time‐series depicted in Figure 5e illustrates the initial formation of the reconstructed edge (State II) resulting from e‐beam induced etching. As the e‐beam etches the phosphorene sample, the exposed edge transitions into State II, as evidenced by the emergence of stacking displacements in Figure 5e. This observation is consistent with previous studies, indicating that State II is energetically favored, being 2.59 eV nm^−1^ lower in energy compared to the pristine edge, State I.^[^ ^90^ ^]^

Once formed, State II remains remarkably stable under e‐beam irradiation, as evidenced by the time‐series in Figure 5f,g. Prolonged irradiation may lead to the formation of local defects and associated stacking displacement at the edge, as illustrated in Figure 5g. These edge defects likely result from the formation of local vacancies and the breaking of interlayer bonds. Notably, these defects tend to self‐heal, with the edge reverting to State II. Employing time‐dependent edge displacement analysis, we estimated the lifetime of local defects at zigzag terminations (Figure S9, Supporting Information). Starting with the local formation of State I, we analyzed its time‐dependent occupancy (Figure S10, Supporting Information) and noted a gradual decrease in State I occupancy over time. Figure 5h reveals that the time dependence of State I occupancy follows an exponential decay, with a temporal decay parameter of approximately 0.43 s.

## 3 Conclusion

Our study showcased the efficient identification of stacking displacements in bilayer phosphorene using a deep learning model. The model accurately detects stacking displacements with an error margin of 3.3% within the unit cell and achieves spatial resolution that approaches the scale of individual unit cells. Applied to a broad dataset of in situ TEM data, the model effectively captured the spatially varying, time‐dependent interlayer displacement dynamics associated with edge reconstruction, processing approximately 9 million atoms across 739 images in roughly 12 min. Our approach not only offers superior spatial resolution compared to existing methods for phase‐contrast TEM imaging but also facilitates high throughput processing of in situ TEM data. The methodology can be extended to other bilayer systems using a similar training process, provided that the symmetry of the unit cell and stacking configurations are properly considered. Potential applications also include twisted layered systems and dynamic phenomena such as interlayer sliding. The ability to precisely analyze layer displacements offers deeper insights into their roles in electrical, mechanical, ferroelectric, and other strongly correlated phenomena of vdW materials.

## 4 Experimental Section

### Sample Preparation

Phosphorene/graphite vertical heterostructures were fabricated using a dry transfer technique within an inert atmosphere glovebox, where concentrations of oxygen and water vapor were maintained below 0.1 ppm. Phosphorene flakes (sourced from Smart Elements) and graphite were mechanically exfoliated onto polydimethylsiloxane (PDMS) supports. Graphene flakes, comprising fewer than 5 layers were transferred onto heating E‐chips (Protochips, Inc.). The thickness of these flakes was predetermined using a DM‐750 M optical microscope (Leica) in transmission mode. These samples were then placed on a Fusion in situ heating holder (Protochips, Inc.) and heated to 500 °C for 3 h under a vacuum of approximately 10^−7^ Torr to eliminate surface residues from the graphene flakes. Few‐layer phosphorene flakes, consisting of up to five layers, were transferred onto the graphite flakes employing the same method. Further details are described in previous literature.^[^ ^90^ ^]^

### Transmission Electron Microscopy and Simulation

TEM images were acquired using a JEOL‐ARM 200F TEM microscope with a double Cs‐aberration corrector operating at 80 kV. The vacuum level within the specimen column was maintained at approximately ≈10^−7^ mbar. In situ observation of the samples at elevated temperatures was conducted using a Fusion in situ holder. During TEM imaging, the samples were heated at a rate of 1 °C s^−1^ and maintained at 270 °C. Most of the TEM time‐series images were acquired with an exposure time of 0.16 s, under an electron dose rate of approximately 2.0  ×  10^6^ e^−1^nm^−2^ s^−1^. TEM image simulations were performed using abTEM software, with parameters set to *C*_s_ = −10 µm, a convergence angle of 0.10 mrad, an objective aperture radius of 1.5 Å^−1^, and mechanical vibration of 0.5 Å (or equivalent simulation conditions) at various defocus values. During the experiment, phosphorene samples were subjected to heating and layer‐by‐layer etching.^[^ ^90^ ^]^ This process was carefully monitored to ensure the formation of bilayer phosphorene regions.

### Deep Learning Model and Statistical Analysis

A residual convolutional neural network^[^ ^60^ ^]^ (ResUNet) with 5 residual blocks for downsampling and upsampling tasks was employed. A total of 40960 simulated images, varying in defocus, crystal angle, stacking configuration, and noise levels, were used to enhance accuracy and prevent overfitting. The model underwent training for 2000 epochs processing a randomly selected set of 1536 images per epoch using a batch size of 32. A detailed description of the deep learning process is provided in Note S2 (Supporting Information). In‐house Python codes were used for all statistical analyses and programming tasks. In data analysis, the least squares method was applied for fitting. All images were normalized to values between 0 and 1, and statistical results were expressed as mean ± standard deviation.

## Conflict of Interest

The authors declare no conflict of interest.

## Supporting information



Supporting Information

Supplemental Movie 1

## Data Availability

The data that support the findings of this study are available from the corresponding author upon reasonable request.
