# Supplementary material for: Deep Learning Analysis of Localized Interlayer Stacking Displacement and Dynamics in Bilayer Phosphorene
Source: Adv Mater. 2025 Mar 3;37(14):2416480. doi: 10.1002/adma.202416480 (PMC11983260; doi:10.1002/adma.202416480)
Supplement: Supplementary file 1 — Supporting Information [file ADMA-37-2416480-s002.docx]

**Supporting Information for**

**Deep Learning Analysis of Localized Interlayer Stacking Displacement and Dynamics in Bilayer Phosphorene**

Kihyun Lee^1,2^, Sol Lee^1,2^, Yangjin Lee^1,3,*^_,_ and Kwanpyo Kim^1,2,*^

^1^Department of Physics, Yonsei University, Seoul 03722, South Korea

^2^Center for Nanomedicine, Institute for Basic Science, Seoul 03722, South Korea

^3^Department of Energy Science, Sungkyunkwan University, Suwon 16419, South Korea

* Corresponding authors: Y.L ([yangjinlee@skku.edu](mailto:yangjinlee@skku.edu)) and K.K. ([kpkim@yonsei.ac.kr](mailto:kpkim@yonsei.ac.kr))

**Supporting Note 1. Interlayer displacement analysis based on geometric phase analysis (GPA)**

The intensity in TEM image is expressed as $I\left( \boldsymbol{r} \right)= \sum_{g} H_{g}\left( \boldsymbol{r} \right)e^{2\pi i\boldsymbol{g}\cdot\boldsymbol{r}}$, where $\boldsymbol{r}$ represents position, $\boldsymbol{g}$ denotes the Bragg reflections in *k*-space, and $H_{g}\left( \boldsymbol{r} \right)$ represents the Fourier component coefficients. If there is slight strain in atomic lattice, it induces changes in $\boldsymbol{g}$ value, $\Delta\boldsymbol{g}$, which causes a shift in the phase of the corresponding sigma component and change in the local intensity $I\left( \boldsymbol{r} \right)$. By differentiating $I\left( \boldsymbol{r} \right)$ with respect to $\boldsymbol{r}$, phase retrieval can be performed in GPA.

In the case of interlayer displacements, the variation in $H_{g}$ is the dominant factor while the change in $\boldsymbol{g}$ is minimal. As a result, phase changes cannot be retrieved through differentiation, making GPA unsuitable for analyzing the interlayer displacements. Figure S1 shows GPA results applied to our experimental data of bilayer phosphorene. The GPA process is found to be inadequate to properly identify the interlayer displacement near the edge.

**Supporting Note 2. Generation of simulation images and deep learning model training**

We created an FCN model that identifies layer displacement of bilayer phosphorene, based on the ResUNet model structure. The model was trained using 40,960 images (256 $\times$ 256 pixels) with varying defocus and signal-to-noise ratio (SNR) levels. The defocus values (-8 nm, -6 nm, and 14 nm) were chosen to encompass both experimental conditions and contrast inversion in phase-contrast imaging. The various stacking configurations were systematically produced by either applying strain (0, 0.002, 0.005, 0.01, 0.02, 0.03, 0.05 and 0.1) to one layer or rotating one layer (0.1$^{\circ}$, 0.2$^{\circ}$, 0.3$^{\circ}$, 0.4$^{\circ}$, 0.5$^{\circ}$, 1$^{\circ}$, 2$^{\circ}$, 3$^{\circ}$, 4$^{\circ}$ and 5$^{\circ}$), resulting in the locally-varying stacking shifts in bilayer systems. This process resulted in a total of 222 unique images, calculated as 3 (defocus) × (8 × 8 strain combinations + 10 rotations). We also performed random rotations on the produced images and cropping to generate diverse image set. Additionally, Poisson shot noise was introduced, with an average number of events *N* ranging from 1,000 to 10,000 [See Figure S3 and S4].

The data were split using a 4:1 train/validation split ratio. We used the Adam optimizer with a learning rate of 0.01, and reduced mean loss function. During training, a dropout rate of 0.5 was applied, with a batch size of 32 for each training step.

Since supervised deep learning model must satisfy the condition that ${f(x}_{1})\neq f\left( x_{2} \right)\to x_{1}\neq x_{2}$, labeling the displacement in the armchair and zigzag directions as (dx, dy) becomes challenging. For instance, $\lim_{n\to\frac{1}{2}} (n, 0)$ and $\lim_{n\to\frac{1}{2}} (0, n)$ correspond to AA and AC stacking configurations, which appear identical in plan-view TEM observation. Due to this reason, the model encounters ambiguity during training, as there are two possible correct answers: (1/2, 0) and (0, 1/2). Moreover, the nature of phase contrast imaging presents a challenge in model training when using actual displacements along the zigzag and armchair directions as labels.

To address this issue, we utilized structural symmetry for labeling layer displacements. In Figure S2, for cases where *dx + dy* ≥ 1/2, we created new labels by symmetrizing with respect to the point (1/4, 1/4). Additionally, to treat AC stacking and AA stacking equivalently, we applied symmetrical labelling with respect to the $dx=dy$ line, as described in Figure S2b. This process identifies the displacement values along major and minor axes, without specifying whether they corresponded to the armchair or zigzag directions.

In our study, the comparison with experimental data was performed to discern whether the major axis corresponds to armchair or zigzag directions. As shown in Figures 3e-3j, when simulating TEM images based on the major/minor displacement values predicted by the model, we observed that the major displacement corresponds to the displacement along the armchair direction. This is also consistent with our expectation that the major displacement occurs perpendicular to the edge termination direction, which is zigzag termination. Although occasional reversals of major and minor displacements may occur in regions with minor displacements away from the edge, these deviations were typically within a 2% range [See Figure S2a]. This finding is further supported by the results presented in Figures 3e~3j. For model applications toward experimental data, we divided images into 256×256 patches with a stride of 16 for significant overlap and removed the edge regions to minimize errors. The evaluated patches were then merged back into the full image.

To verify whether the generated data encompasses the actual experimental data, we conducted PCA (Principal Component Analysis). Figure S5a shows the PCA results for the training data, test data, and experimental data. We found that the training data represents a broader range of environments compared to the actual experiments. For PCA1, we identify the strong correlation between PCA1 with the defocus values of the images as shown in Figure S5b. On the other hand, PCA2, which has a more minor effect, shows little visible variation and does not reveal concrete physical interpretation at this point.

**Supporting Note 3. Analysis of electron diffraction and Fourier transformations**

The structure factor $F$ is given by the following equation,

|  | $F_{hkl}=\sum_{j=1}^{N} f_{j}(\vert\vec{G}\vert)e^{[-2\pi i\left( hx_{j}+ky_{j}+lz_{j} \right)]}$ | (1) |
| --- | --- | --- |

where $f_{j}$ represents the atomic form factor, (*hkl*) denotes the Miller index as arbitrary integer numbers, and ($x_{j}$,$y_{j}$,$z_{j})$ represents the basis atom $j$ position, and $\vec{G}$ is the reciprocal lattice. To investigate the intensity variations of the (200), ($1\bar{1}0$), and (020) peaks in bilayer phosphorene based on its stacking configuration, electron diffraction simulations were conducted. The atomic form factor is approximated by a sum of Gaussians, as expressed by the formula $f_{j}\left( \left| \vec{G} \right| \right)= \sum_{i=1}^{4} a_{i}e^{-b_{i}\left( \frac{G}{4\pi} \right)^{2}}+c$. The coefficients for each term are adopted from a reference^[1]^. Since the scattering intensity is the square of the Structure factor, it can be expressed as follows.

|  | $I_{hkl}\left( u,v \right)=F_{hkl}^{*}\times F_{hkl}=\left\vert f_{j}\left( \left\vert\vec{G} \right\vert\right) \right\vert^{2}\sum_{i,j=1}^{N} e^{[-2\pi i\left( hx_{ji}+ky_{ij}+lz_{ij} \right)]}$. | (2) |
| --- | --- | --- |
|  | $p_{ji}\equiv p_{j}-p_{i} (p=x, y,z)$ | (3) |

Let's denote the real-space image intensity as $I\left( x,y \right)$. The Fourier transformation relation can be written as

|  | $F\left( u,v \right)=\int_{-\infty}^{\infty} \int_{-\infty}^{\infty} I\left( x,y \right)e^{-2\pi i(ux+vy)}dxdy$ | (4) |
| --- | --- | --- |

and its inverse relationship

|  | $I\left( x,y \right)=\int_{-\infty}^{\infty} \int_{-\infty}^{\infty} F\left( u,v \right)e^{2\pi i\left( ux+vy \right)}dudv$ | (5) |
| --- | --- | --- |

When comparing equations (2) and (5), it is evident that the peak intensity in the electron diffraction pattern is identical to the Bragg peak intensity obtained from the Fourier transform of the measured atomic-scale image. This is expressed by the square of the Structure factor. The Debye-Waller factor due to thermal vibration and the sampling size used in Fourier transform are both factors that affect peak intensity. However, since the correction is done by convolving additional variables with the existing peak, the absolute intensity values may change, but the relative intensity values between peaks remain unaffected. Therefore, we conducted intensity analysis without noise. In Figure S6, the square of the structure factor for phosphorene according to layer displacements was theoretically calculated. Figure S6j shows the calculation of the structure factors $S_{200}$, $S_{1\bar{1}0}$ and $S_{020}$ for each position in the image with various stacking configurations in Figure 1. Cropping the image to the size of two-unit cells and performing a Fourier transform yields the same results for the intensity of each Bragg peak.

**Supporting Note 4. Spatial resolution of deep learning-based displacement identification**

We compare the spatial resolution from the conventional Fourier transform-based method and the deep learning model. Figure S7a shows a bilayer phosphorene TEM image with a clearly visible edge. The model's predictions and the intensity representation using the Bragg filter method are shown in Figure S7b and S7c, respectively. In Figure S7c, the Bragg filter method introduces phantom atoms as artifacts due to its Fourier transform-based approach. We line-profiled predictions from the edge to the vacuum region (Figure S7d). The deep learning model and Bragg-filtering method exhibit full width at half maximum (FWHM) values of approximately 3.7 Å and 9.4 Å, respectively. When normalizing the line profile results from the phosphorene edge to bulk regions, the layer displacement decaying rate is also faster in the case of deep learning model, demonstrating the higher spatial resolution of deep learning method. Upon applying a Gaussian blur to the model-predicted data, the resulting graph gradually aligns with that from the Bragg filter method, as shown in Figure S7e. With a sigma value of approximately 7.4 Å for Gaussian blur, the results from the artificial intelligence model and the Bragg filter method become nearly identical [See Figure S7f]. 7.4 Å corresponds roughly to the double unit-cell size of phosphorene and the estimated spatial resolution of Bragg filter-based method.

**Supporting Note 5. Edge-associated energy analysis**

**1) Strain energy**

In the linear elastic material model, strain ($\epsilon$) and stress ($\sigma$) are expressed as the matrix-tensor product of the elasticity matrix (*D*) as follows:

|  | $\boldsymbol{\sigma}=D\boldsymbol{:\epsilon}$ | (6) |
| --- | --- | --- |

where *D* is symmetric 6-by-6 matrix.

When assuming a plate of very thin thickness and considering an orthotropic model, the stress-strain relation is expressed as follows:

|  | $\left( \begin{matrix} \begin{matrix} \\ \epsilon_{xx} \\ \end{matrix} \\ \epsilon_{yy} \\ \begin{matrix} \\ \epsilon_{xy} \\ \end{matrix} \end{matrix} \right)=\left( \begin{matrix} \frac{1}{E_{x}} & -\frac{\nu_{y}}{E_{y}} & 0 \\ -\frac{\nu_{x}}{E_{x}} & \frac{1}{E_{y}} & 0 \\ 0 & 0 & \frac{1}{G_{xy}} \end{matrix} \right)\left( \begin{matrix} \begin{matrix} \\ \sigma_{xx} \\ \end{matrix} \\ \sigma_{yy} \\ \begin{matrix} \\ \sigma_{xy} \\ \end{matrix} \end{matrix} \right)$ | (7) |
| --- | --- | --- |

with Young's modulus ($E_{x}$ and $E_{y}$), shear modulus ($G_{xy}$), and Poisson's ratios ($\nu_{x}$ and $\nu_{y}$) in the Cartesian system^[2-3]^.

The strain energy can be calculated as follows:

| $E_{strain}=\frac{1}{2}\int\left( E_{x}{\epsilon^{2}}_{xx}+E_{y}{\epsilon^{2}}_{yy}+G_{xy}{\epsilon^{2}}_{xy} \right)dV$ | (8) |
| --- | --- |

The layer displacement values are extracted by the deep learning model [See Figure 4b-4c] and the strain can be extracted from the displacement vector (**u**) as follows:

| $\boldsymbol{\epsilon}=\frac{1}{2}\left[ \boldsymbol{\nabla u}+\left( \boldsymbol{\nabla u} \right)^{T} \right]$ | (9) |
| --- | --- |

Figure 4d-4f depicts the visualization of strain in each direction calculated using equation (9). If we assume the one-dimensional stain field perpendicular to the edge termination, the average strain energy stored per unit length along the zigzag direction is calculated to be approximately 0.04 eV/nm.

**2) Potential energy surface (PES) and interlayer stacking energy**

The relative interlayer stacking energy ($E_{r}$) represents the difference in energy of a certain stacking configuration compared to the most stable state of bilayer phosphorene, AB stacking configuration.

|  | $E_{r}=E_{stacking}-E_{AB}$ | (10) |
| --- | --- | --- |

The Potential Energy Surface (PES)^[4]^ represents the interlayer interaction energy, showcasing how the relative energy changes with layer displacement in vdW materials. We utilized the PES values calculated by Alhassan, A., and Yu, M^[4]^. For instance, in the case of AA stacking, which involves displacing 50% of the unit cell with respect to AB stacking in the zigzag direction, the PES at position (1/2, 0) in Figure S2a exhibits a value of 9.3 meV/atom. We calculated the PES at each position using the layer displacement extracted from images, demonstrating how the stacking changes occurring due to reconstruction become energetically less stable compared to AB-stacked bilayer phosphorene structure [See Figure 4g]. The average stacking energy per unit length ($E_{stacking}$) is found to be 0.25 eV/nm.

**3) Energy competition between strain energy and interlayer stacking energy**

Assuming a simple decaying model for the edge-localized displacement in bilayer phosphorene, the displacement can be expressed as follows.

| $D(y)=Ae^{-y/\tau_{s}}$ | (11) |
| --- | --- |

where function $D(y)$ represents the local layer displacement at position *y*, *A* represents the displacement amplitude, and $\tau_{s}$ represents the spatial decaying parameter. Let us denote the mapping that transforms an arbitrary layer displacement vector $\vec{r}=\left( x,y \right)$ into stacking interaction energy as *M*($\vec{\boldsymbol{r}}$) :

($\vec{\boldsymbol{r}}\underset{\to}{M}PES\left( \vec{\boldsymbol{r}} \right) = M\left( \vec{\boldsymbol{r}} \right))$.

The mapping *M* represents the potential energy surface (PES). The change in stacking interaction energy per unit length, $E_{stacking}$, is given by the following equation,

| $E_{stacking}\mathbf{=}\int PES\left( D\left( y \right) \right)dy =\int_{0}^{\infty} M\left( Ae^{-y/\tau_{s}} \right)dy$ | (12) |
| --- | --- |

The *y*-directional strain $\epsilon_{yy}$ are obtained from equations (10) and (12) as follows.

| $\epsilon_{\mathrm{yy}}=\frac{\partial D}{\partial y} = \frac{\partial}{\partial y}(Ae^{-y/\tau_{s}}) = -\frac{A}{\tau_{s}}e^{-y/\tau_{s}}$ | (13) |
| --- | --- |

By assuming that there is no tensile strain or shear strain along the zigzag direction, the strain energy per unit length $E_{strain}$ is obtained from equation (8) as follows.

| $E_{strain}=\frac{1}{2}\int E_{y}{\epsilon^{2}}_{yy}dy=\frac{1}{2}\int_{0}^{\infty} E_{y}\left( -\frac{A}{\tau}e^{-y/\tau_{s}} \right)^{2}dy = \frac{E_{y}A^{2}}{4\tau_{s}}$ | (14) |
| --- | --- |
|  |  |

By combining equations (12) and (14), we obtain the total energy associated with interlayer stacking and strain energy.

| ${\Delta E}_{total} = E_{stacking} + E_{strain}\boldsymbol{=}\int_{0}^{\infty} M\left( Ae^{-y/\tau_{s}} \right)dy + \frac{E_{y}A^{2}}{4\tau_{s}}$ | (15) |
| --- | --- |

The values of ***A*** and $\boldsymbol{\tau}_{\boldsymbol{s}}$ that minimize ${\Delta E}_{total}$ are determined by satisfying the following equation.

| $\frac{\partial{\Delta E}_{total}}{\partial A}=0$ | (16) |
| --- | --- |
| $\frac{\partial{\Delta E}_{total}}{\partial\tau_{s}}=0$ | (17) |

Transitioning from AB stacking to AC stacking and considering AB stacking as the origin, there is symmetry above and below the y-axis. Therefore, the mapping $M\left( \vec{\boldsymbol{r}} \right)$ must satisfy $M\left( x \right)=M\left( y \right)$. Therefore, we can expand $M\left( y \right)$ as $M\left( y \right)=c_{0}+c_{1}y^{2}+\vartheta\left( y^{4} \right)$, and equation (16) yields a trivial solution, $A=0$. Expanding equations (16) and (18), we obtain the following equation.

| $\frac{\partial{\Delta E}_{total}}{\partial\tau_{s}}\approx\frac{c_{1}A^{2}}{2}-\frac{E_{y}A^{2}}{4\tau_{s}^{2}}=0 \underset{\to}{} \tau_{s}\approx\sqrt{\frac{E_{y}}{2c_{1}}}$ | (18) |
| --- | --- |

Equation (18) explains that the decaying parameter is determined by the competition between the Young’s modulus and the magnitude in change within PES. Figures S8a and S8b illustrates the variation in total energy ${\Delta E}_{total}$ and optimal decay parameter $\tau_{s}$ with varying Young’s modulus. This calculation obtained numerically by integration exhibits a same trend as equation (18). The theoretical decaying parameter was calculated as 16.3 $Å$ with the theoretical Young’s modulus of phosphorene 43.7 GPa^[2]^, which agrees well with experimentally observed decaying parameter $19.2\pm3.8 Å$.


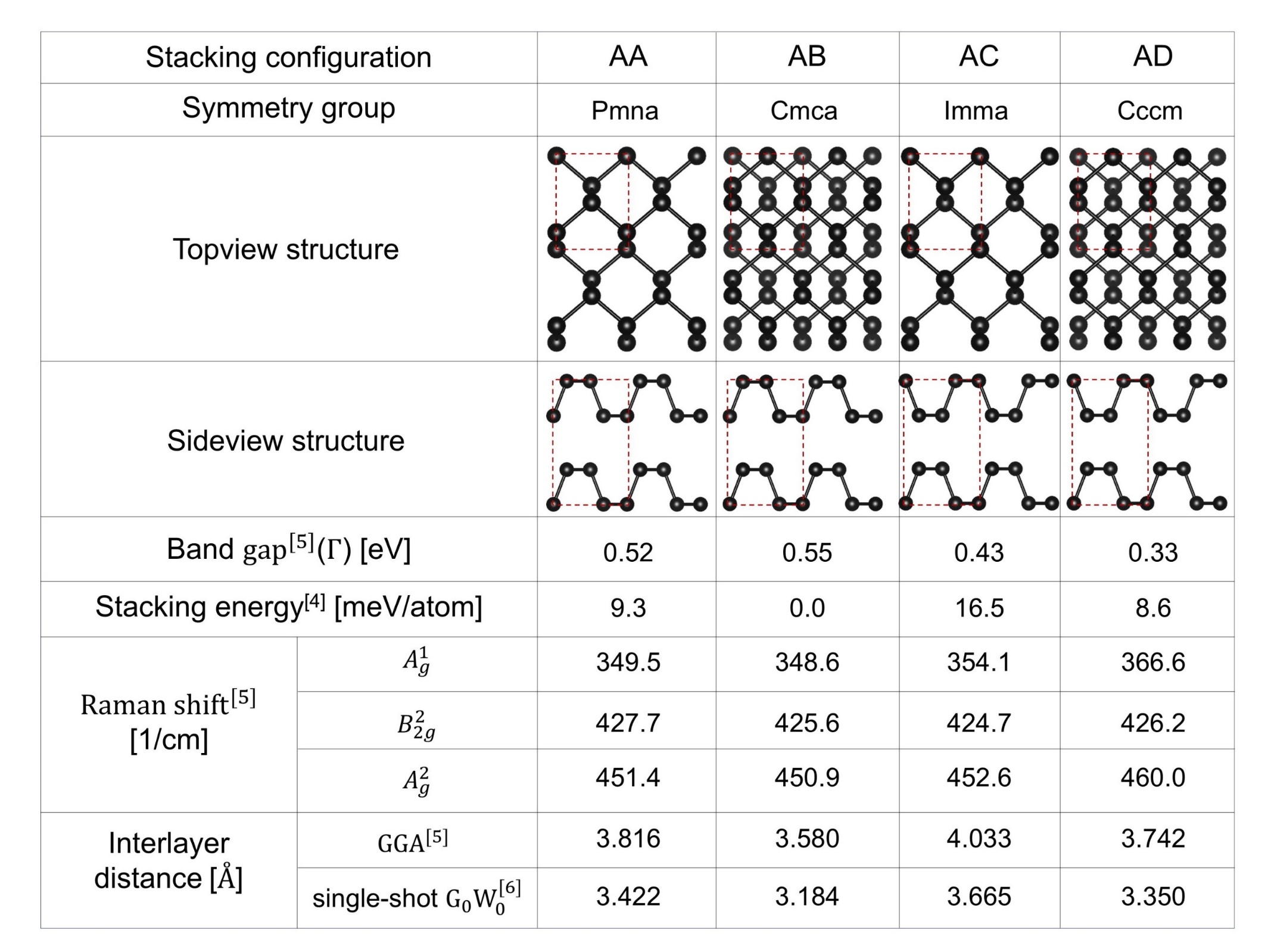


**Table S1. Physical properties of bilayer phosphorene depending on stacking configurations.** The values are adapted from references ^[4-6]^


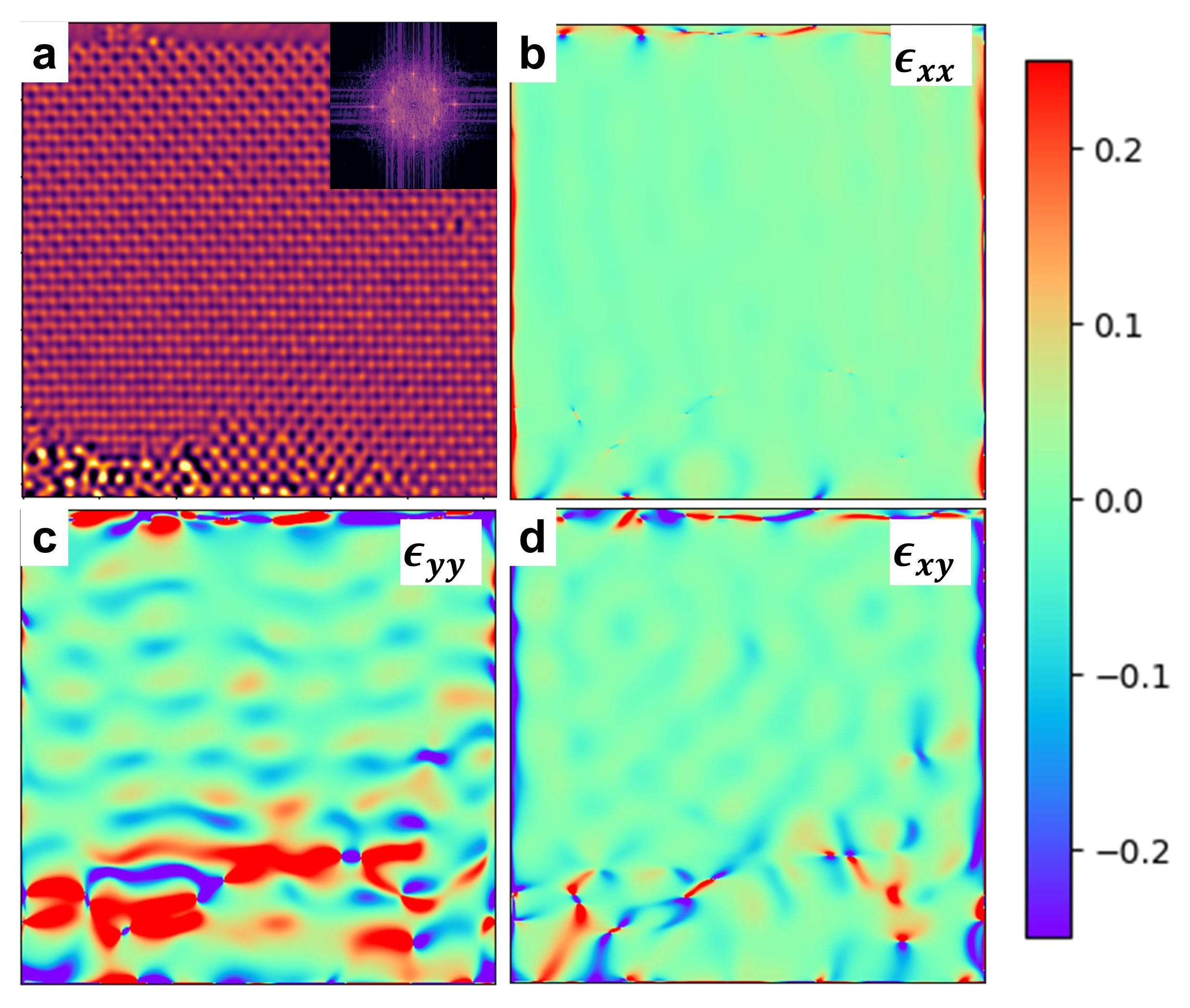


**Figure S1. Geometric phase analysis (GPA) of layer displacement from phase-contrast TEM image of bilayer phosphorene. (a)** Phase-contrast TEM image of bilayer phosphorene sample with edge. **(b-d)** Strain mapping data for the corresponding region, showing strain components, $\epsilon_{xx}, \epsilon_{yy},\mathrm{and} \epsilon_{xy},$ respectively. The localized edge displacement was not adequately identified.


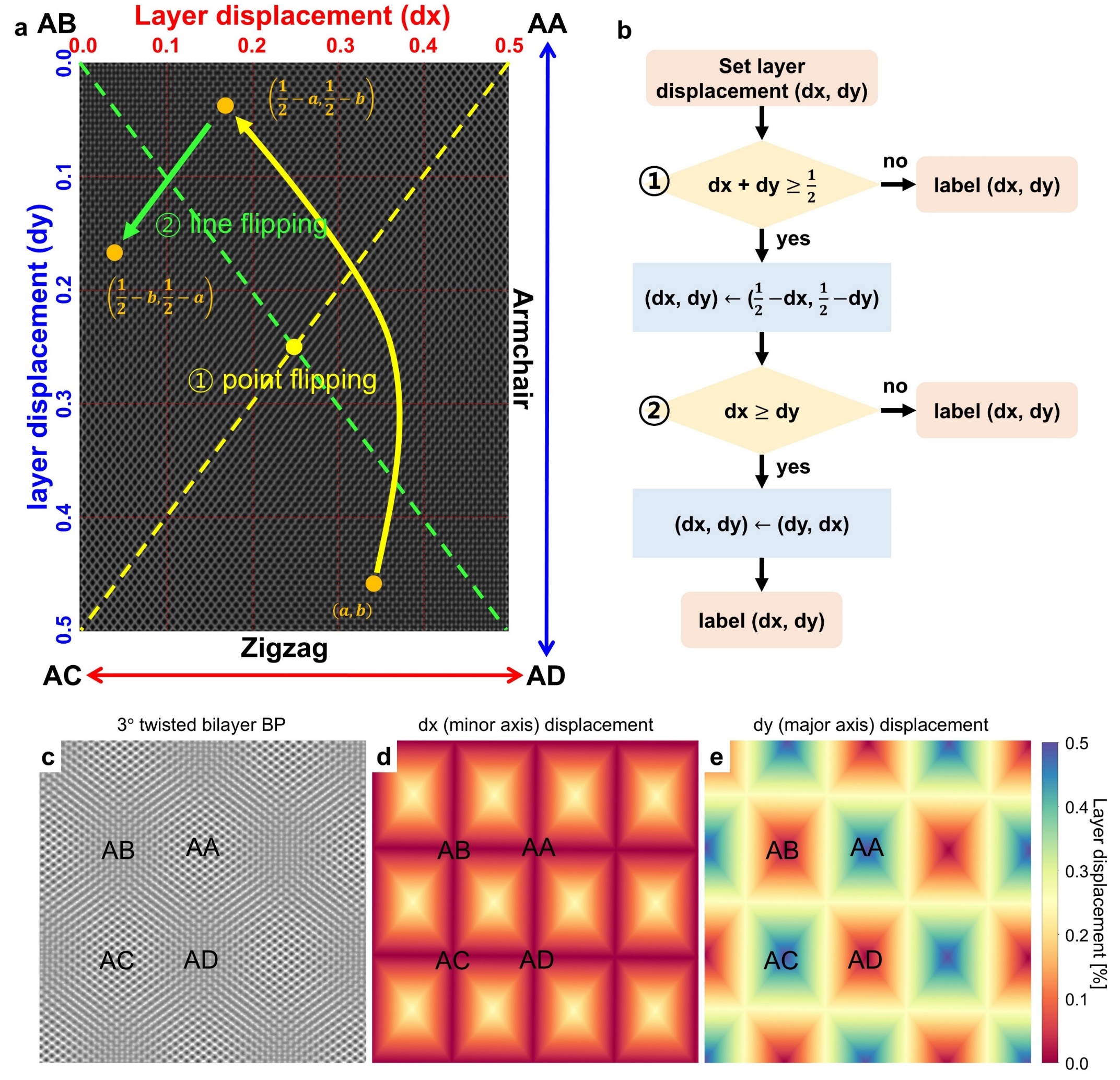


**Figure S2. Method and exemplary images for creating the training dataset. (a**) Schematic diagram illustrating the labeling process for the layer displacement. **(b)** Flowchart depicting the labeling procedure as described in the schematic diagram panel a. **(c)** An example of training data: TEM image of a twisted bilayer phosphorene with a 3-degree rotation. **(d-e)** True labels for the major and minor displacements corresponding to the panel c.


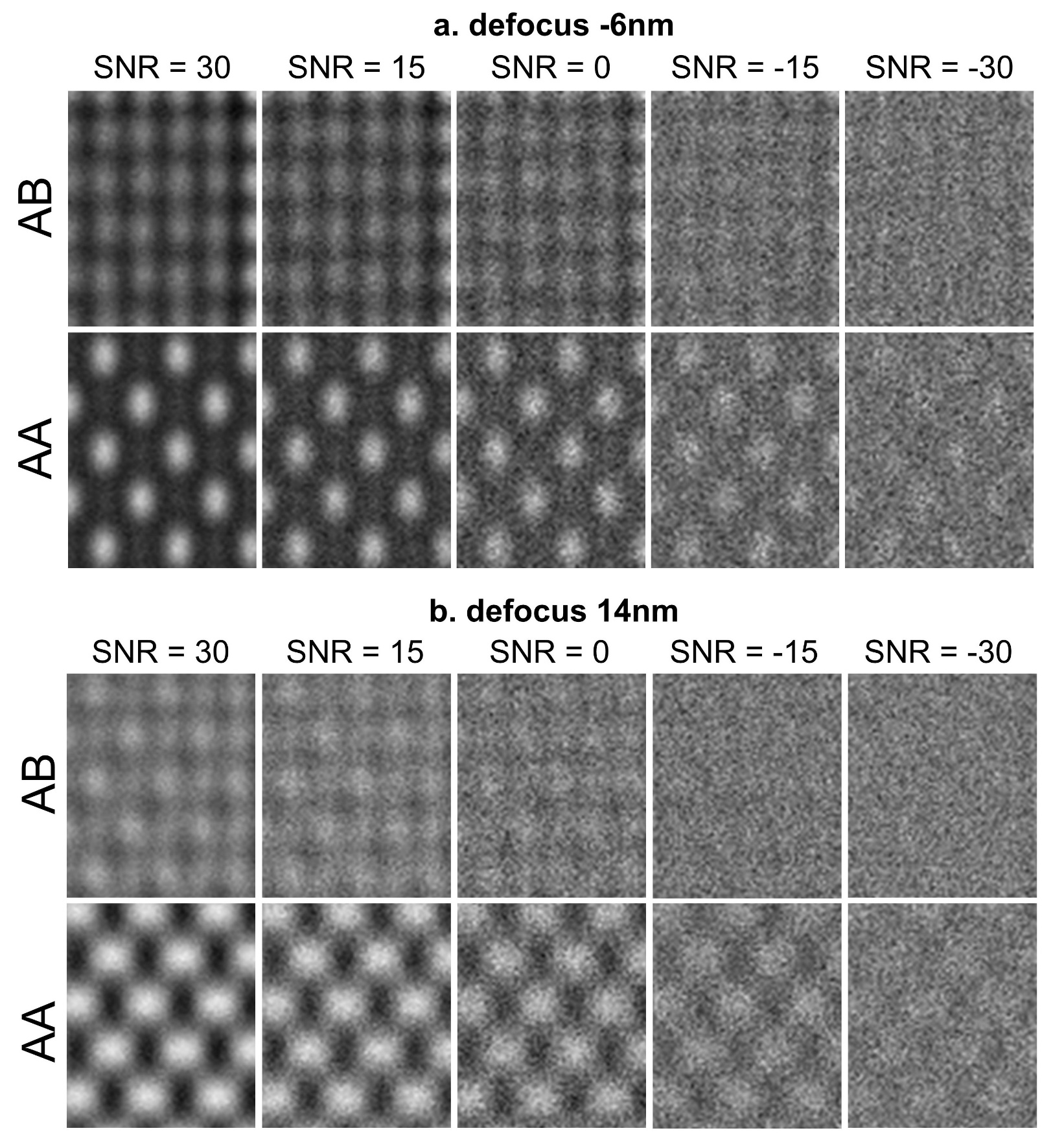


**Figure S3. Simulated TEM images of AB and AA bilayer phosphorene as a function of signal-to-noise ratio (SNR) levels.** Simulated TEM images with various SNR levels at **(a)** -6nm defocus and **(b)** 14nm defocus.


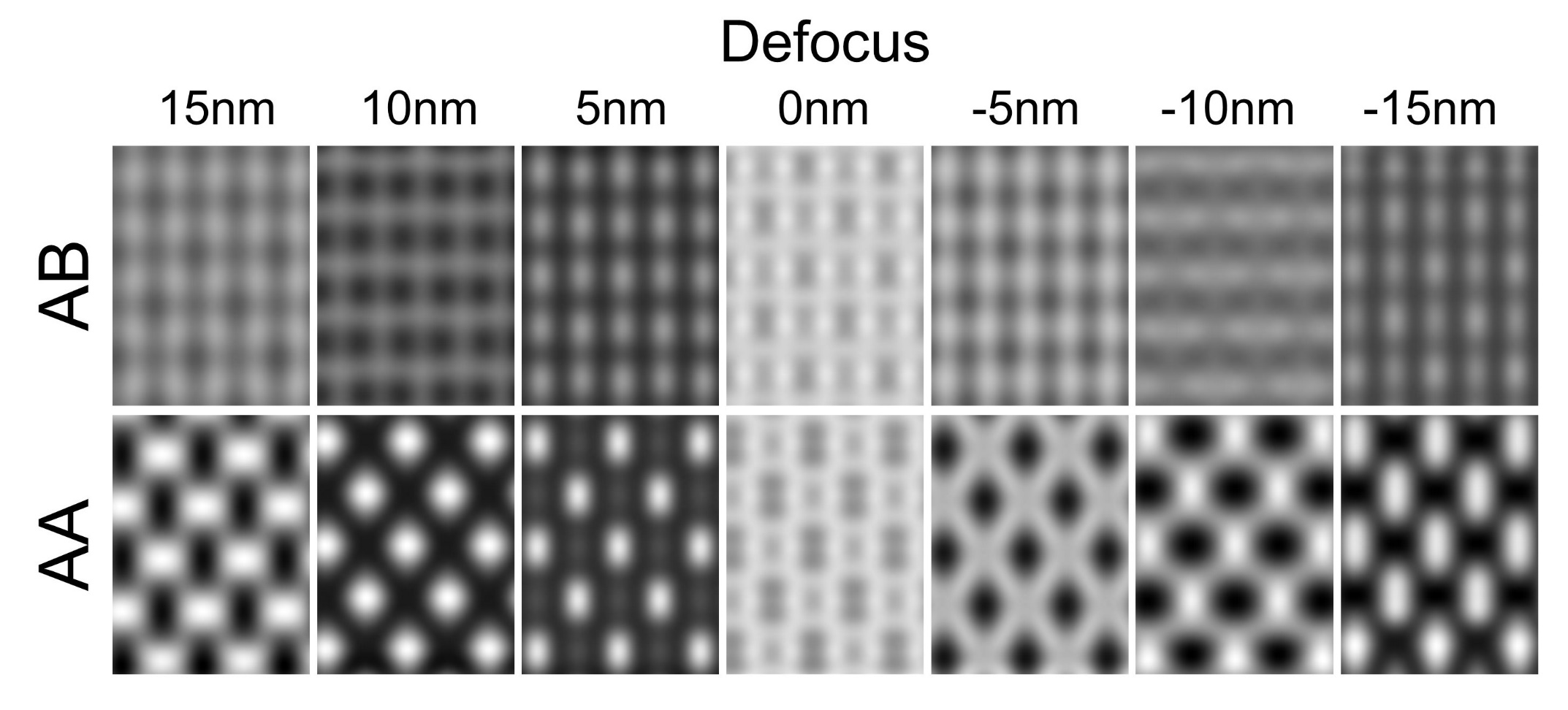


**Figure S4. Simulated TEM images of AB and AA stacking configuration depending on defocus values.**


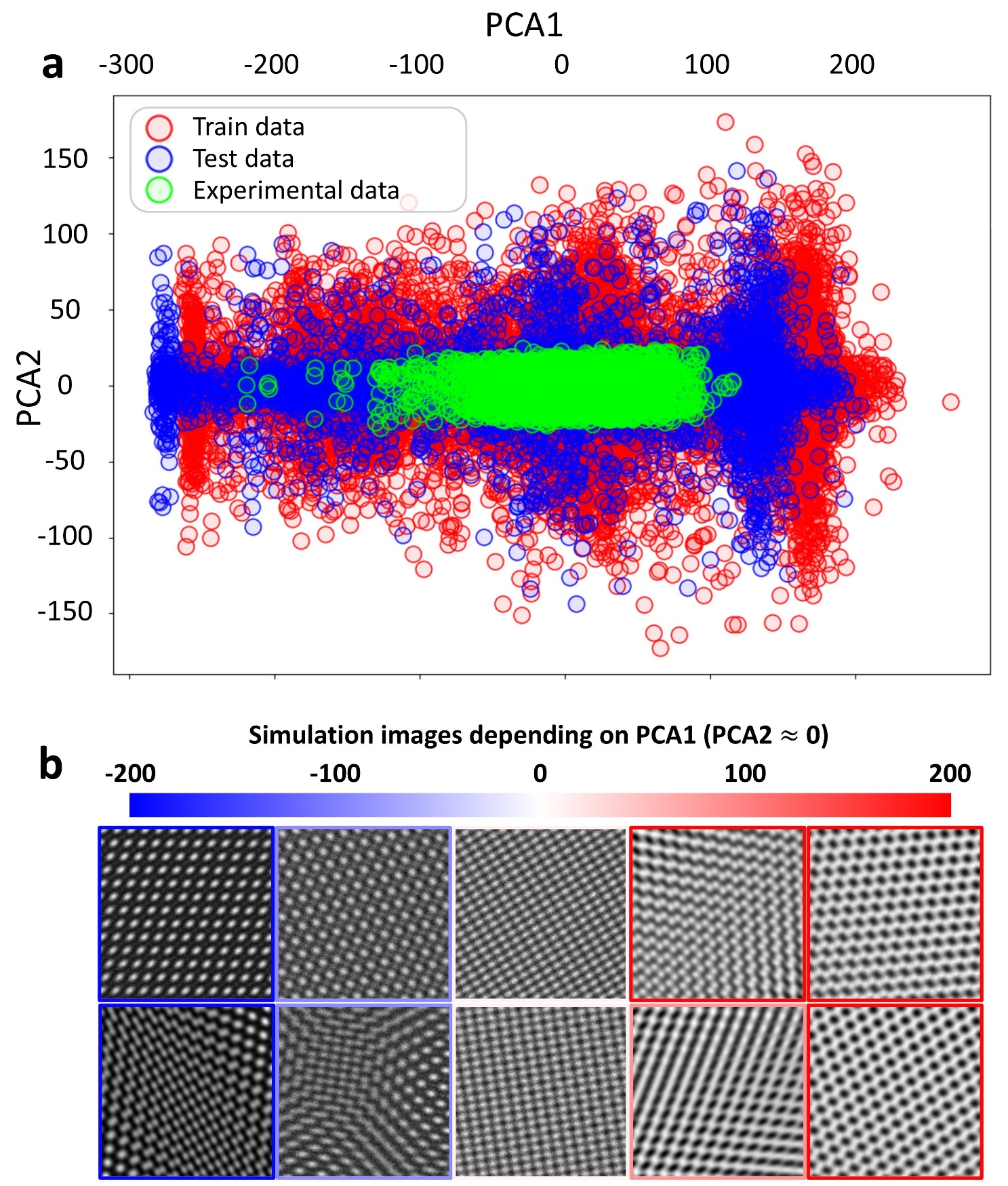


**Figure S5. PCA reduction algorithm (a)** PCA results for train, test, and experimental TEM images projected onto two axes. **(b)** Simulated TEM images showing changes based on PCA1 variations.

**
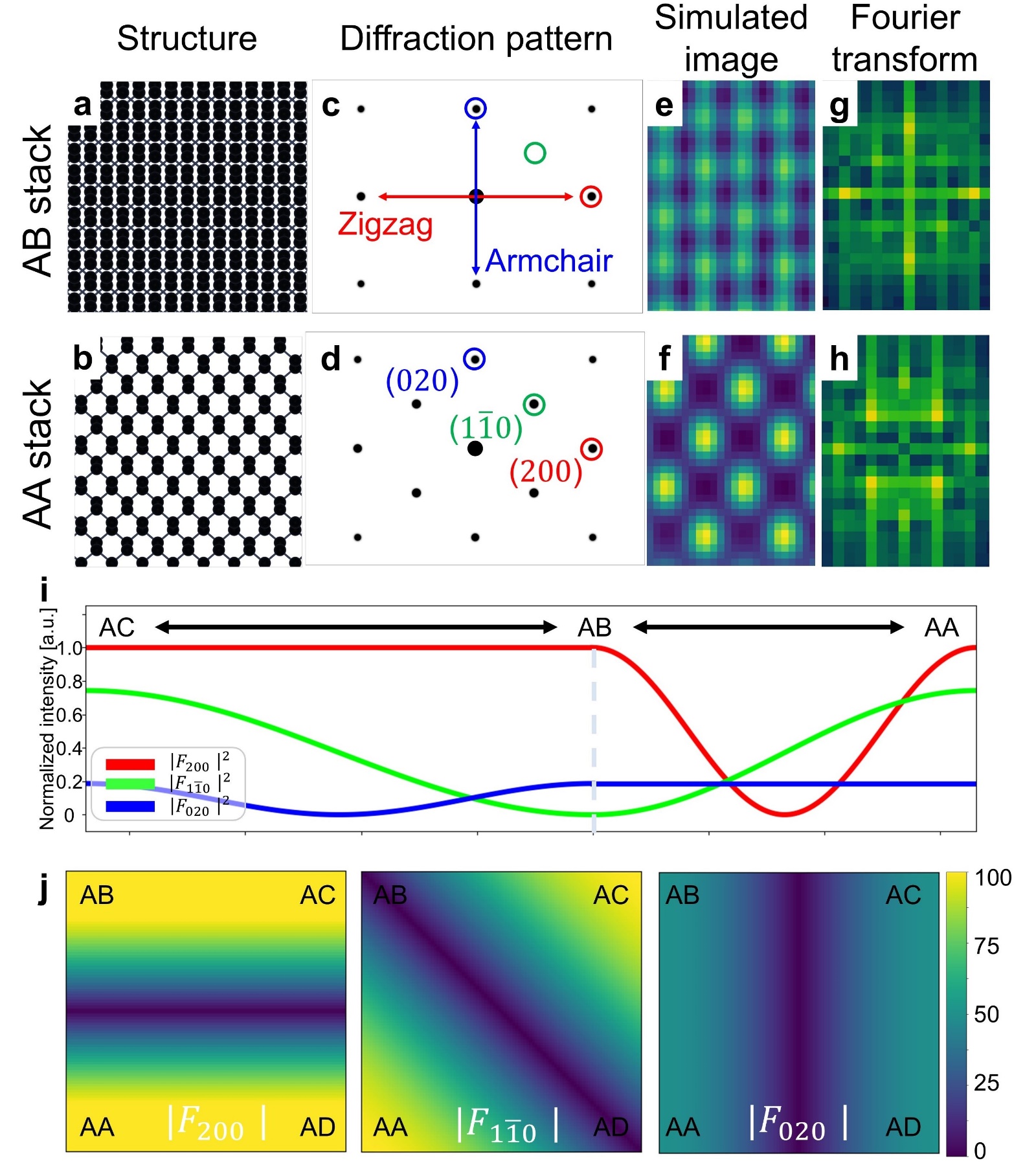
**

**Figure S6. Diffraction intensity analysis of bilayer phosphorene depending on layer displacement values. (a-b)** AB and AA stacking configuration structures. **(c-d)** Simulated electron diffraction patterns for each configuration. **(e-f)** Simulated TEM images and **(g-h)** corresponding FFT images. **(i)** Structure factors corresponding to (020), ($1\bar{1}0$), and (200) peaks as a function of interlayer displacement. **(j)** Structure factor intensity maps with respect to layer displacement along both armchair and zigzag direction.


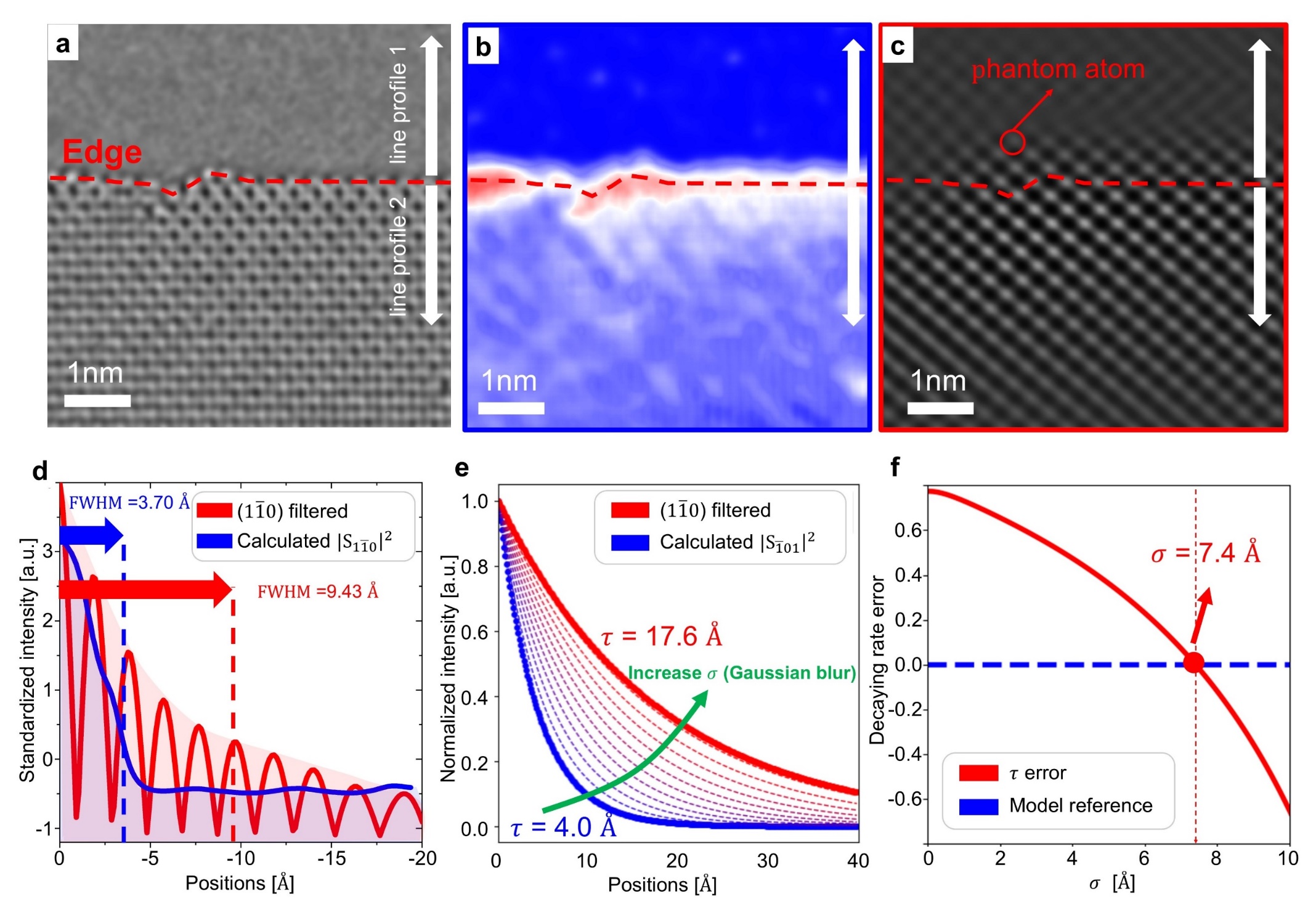


**Figure S7. Spatial resolution comparison between deep learning-based model and Bragg filter method. (a)** TEM image of a bilayer phosphorene with a clearly visible edge. **(b)** Layer displacement results when the image in (a) is processed by the deep learning model. **(c)** Image after applying ($1\bar{1}0$) Bragg filter to the image in (a), showing phantom atoms as artifacts. **(d)** Line profiles from the edge to the vacuum direction for (b) and (c). **(e)** Line profiles from the edge to the bulk for (b) and (c). **(f)** Error in the decay rate between Bragg filter method and the Gaussian-blurred model predictions with a specific standard deviation.


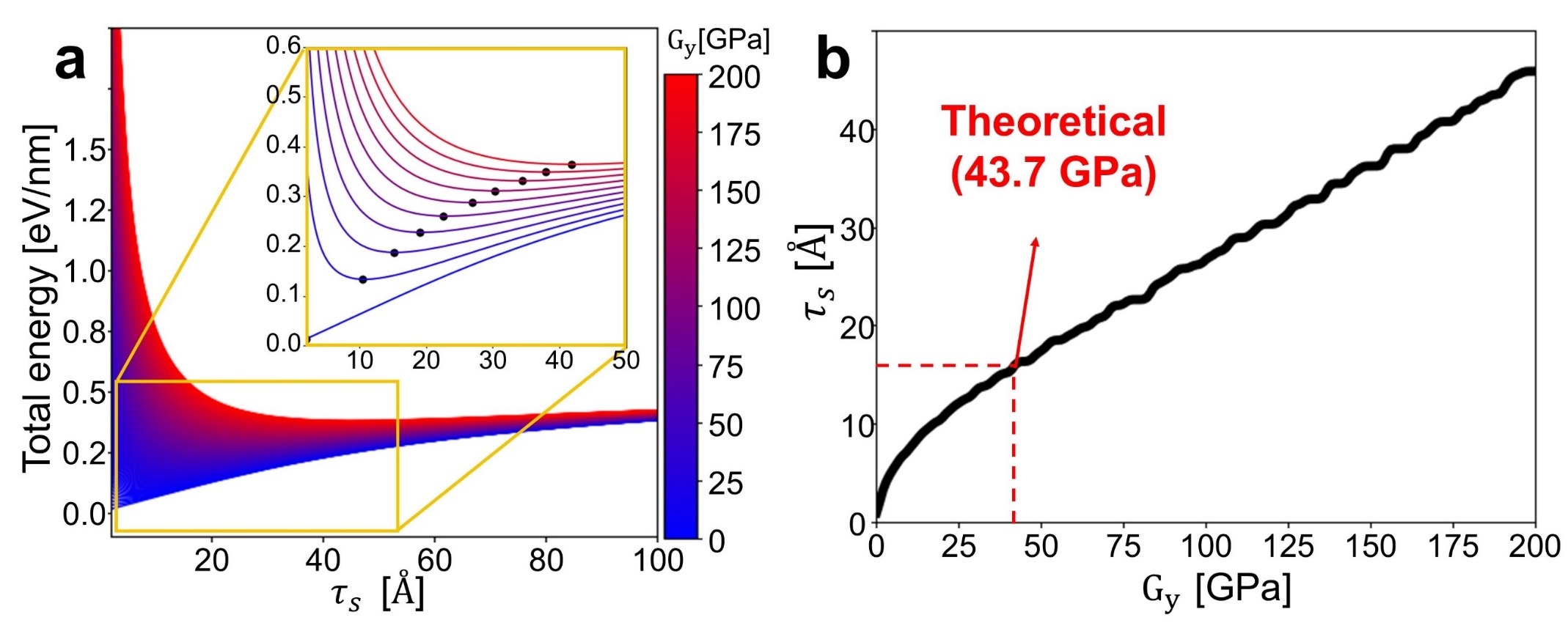


**Figure S8. Decaying parameter analysis based on edge energy. (a)** Total energy variation depending on $\tau_{s}$ at various Young's modulus values. **(b)** Optimal parameter variation according as a function of Young's modulus. The values from the theoretically calculated value is marked.

**
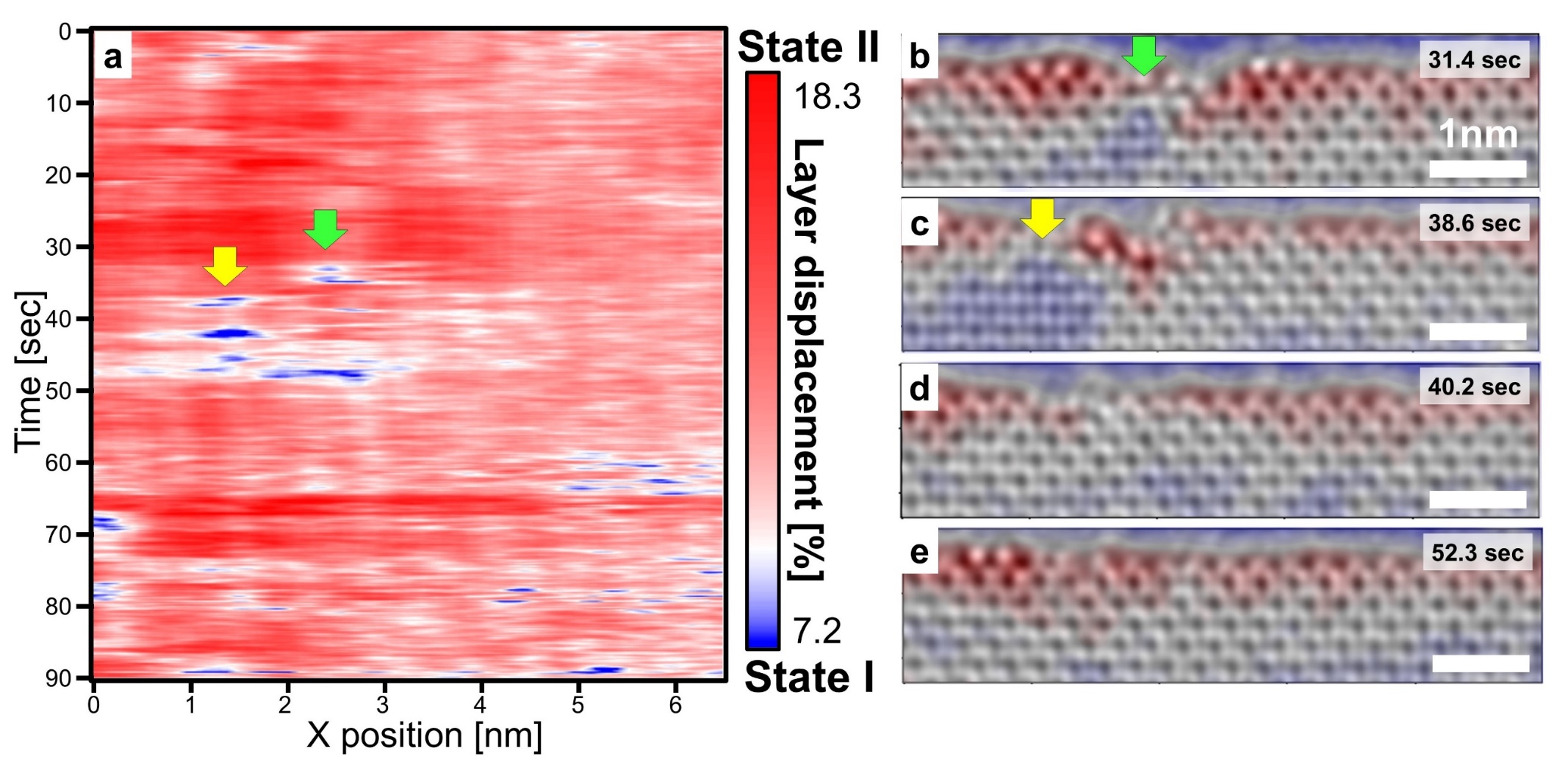
**

**Figure S9. State configuration analysis. (a)** Displacements along *y* direction, $u_{y}$, as a function of time and position. **(b)** Exemplary TEM images with dynamical displacement behavior.


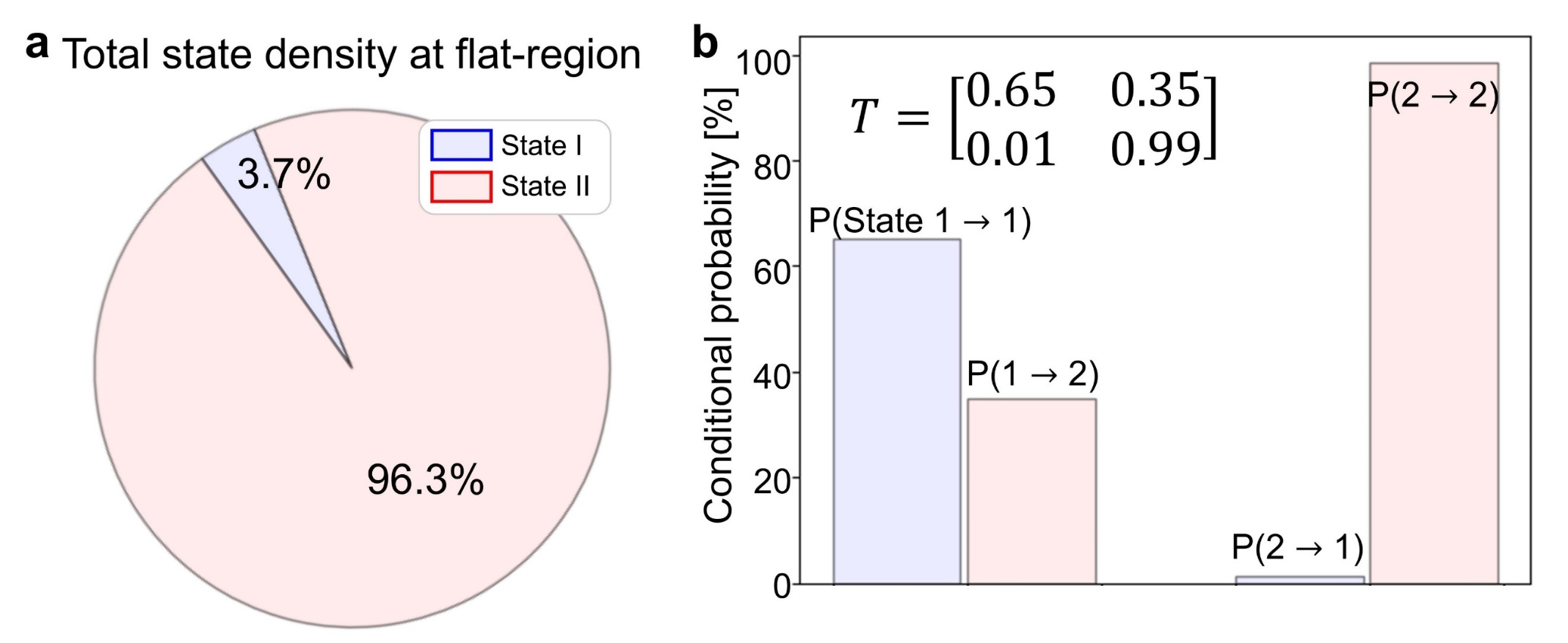


**Figure S10. Statistical analysis of occupied-state probability. (a)** Total state density in the region of flat termination edge. **(b)** Probability of transition rate. The components of the transition matrix $T_{AB}$ represent the probability of transitioning from A state to B state (A, B = State I or State II).

Supporting References

[1] T. Hahn, U. Shmueli, J. W. Arthur, *International tables for crystallography*, Vol. 1, Reidel Dordrecht, **1983**.

[2] Q. Wei, X. Peng, *Appl. Phys. Lett.* **2014**, 104.

[3] J.-W. Jiang, H. S. Park, *J. Phys. D: Appl. Phys.* **2014**, 47, 385304.

[4] A. Alhassan, M. Yu, *Nanotechnol.* **2024**, 35, 155701.

[5] H. Shu, Y. Li, X. Niu, J. Wang, *Phys. Chem. Chem. Phys.* **2016**, 18, 6085.

[6] Y. Wei, F. Lu, T. Zhou, X. Luo, Y. Zhao, *Phys. Chem. Chem. Phys.* **2018**, 20, 10185.
